# A Case of Malignant Metastatic Pheochromocytoma After Eight Years of Primary Diagnosis

**DOI:** 10.14740/wjon760w

**Published:** 2014-03-11

**Authors:** Sakshi Kapur, Afsheen N. Iqbal, Miles B. Levin

**Affiliations:** a Department of Internal Medicine, Overlook Medical Center, Summit, New Jersey 07902, USA; b Division of Medical Oncology, Memorial Sloan-Kettering Cancer Center Basking Ridge, 136 Mountain View Boulevard, Basking Ridge, New Jersey 07920, USA; c Division of Pathology, Overlook Medical Center, Summit, New Jersey 07902, USA

**Keywords:** Pheochromocytomas, Paragangliomas, MIBG, Sunitinib

## Abstract

We report a case of a 66-year-old female who presented to the hospital with abdominal discomfort for 3 months. Work-up revealed a 2.4 cm mass in the right adrenal gland. A laparoscopic resection of the adrenal mass was performed and the histopathology was consistent with a pheochromocytoma. Patient was under active surveillance for 8 years, until she developed local recurrence in the right adrenal bed. A right adrenal bed resection and right nephrectomy were performed. Although the tumor margins were positive, none of the sampled lymph nodes (0/6) were positive for metastasis. Patient refused any adjuvant therapy, and was discharged on surveillance from the hospital. A year later, patient was found to have metastatic disease involving her spine, iliac bones, bilateral hips and right dome of the diaphragm. Patient was offered a metaiodobenzylguanidine scan, and positive subsequent treatment with radioactive iodine was discussed with her. However, she denied any further intervention and was made hospice.

## Introduction

Malignant pheochromocytomas (PCCs) and other paragangliomas are extremely rare tumors. Size greater than 5 cm, extra-adrenal location, “PCC of the adrenal gland scales score” > 6, exclusive dopamine secretion and a Ki-67 index > 3% are potential indicators of malignancy. Although surgery is the mainstay of treatment, in recent years, various other modalities have been used successfully to treat these rare tumors. These include using radioactive iodine (I^123^ and I^131^), external radiation and chemotherapy. A better understanding of the molecular pathways involved in PCCs, and newer treatment options targeting these abnormal signaling pathways, seems to be a promising option for treating these aggressive tumors in future. Our case highlights not only the rarity and the malignant potential of these tumors, but also the challenges encountered in diagnosing and treating these rare tumors. We did extensive search of literature and our case focuses on the histopathological, biochemical, molecular and genetic aspects of malignant PCCs and other malignant paragangliomas.

## Case Report

**Figure 1:**
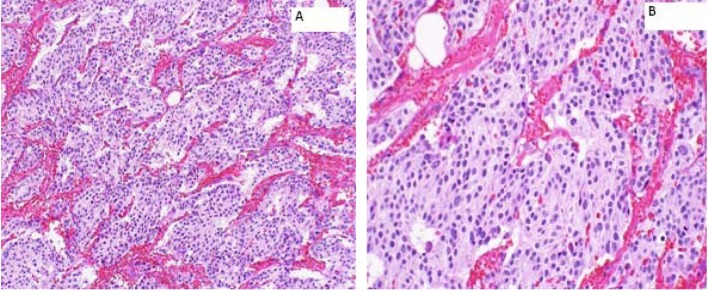
Right adrenal bed resection compatible with recurrent malignant pheochromocytomas. (A) × 100: microscopically, the tumor cells are arranged in well defined nests (“Zellballen”) bound by delicate fibrovascular stroma; (B) × 200: higher power shows amphophilic cytoplasm with a somewhat fibrillary cytoplasm.

A 66-year-old female presented to the hospital in August 2004 with abdominal discomfort for 3 months. Work-up revealed a 2.4 cm mass in the right adrenal gland. However, she denied any headaches, flushing, palpitations or loss of consciousness. A laparoscopic resection of the right adrenal mass was performed and histopathology was consistent with a PCC. Patient was discharged from the hospital on active surveillance and there was no evidence of recurrence of disease till June 2012. However, in November 2012, PET-CT showed recurrence in the tumor bed. A right adrenal mass bed resection and right nephrectomy were performed. Although the margins were positive, none of the sampled lymph nodes were positive (0/6) for metastasis (Fig. 1). Patient refused any adjuvant therapy and was discharged from the hospital on surveillance.

In August 2013, patient presented with pain in her right hip. Physical examination revealed an elderly female in no acute distress. Vital signs were within normal limits except for an elevated blood pressure of 165/98 mmHg. Her karnofsky performance status scale was 70%. Head and neck exam was positive for mild pallor; however, no lymph node enlargement was noted. Both heart and lungs were normal on exam. Abdomen was soft, non-tender with no hepatosplenomegaly. Extremities revealed no clubbing, cyanosis or pedal edema. Both hips were tender on palpation and had decreased range of motion.

**Figure 2:**
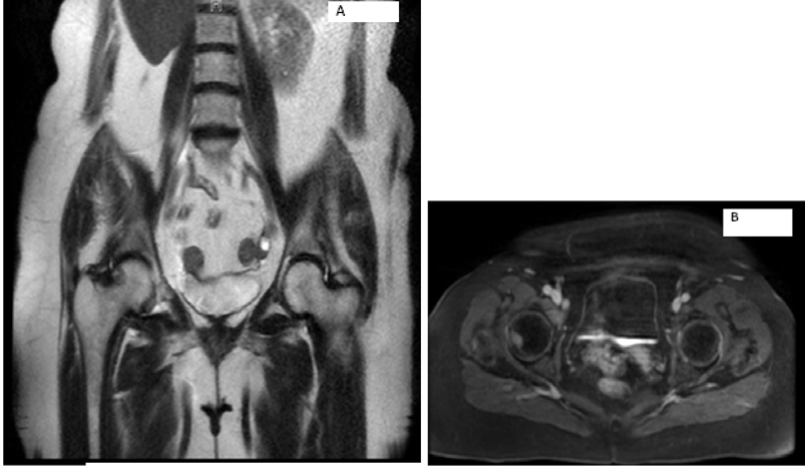
Magnetic resonance imaging of the abdomen showing metastasis to right hip.

**Figure 3:**
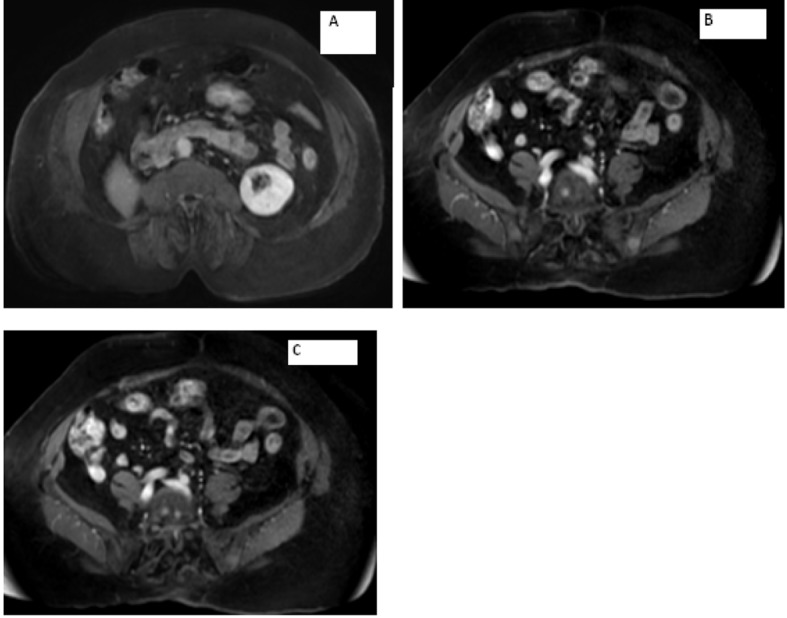
Magnetic resonance imaging of the abdomen showing (A) empty right adrenal and renal fossa and (B, C) metastasis seen in the vertebrae.

Laboratory work-up includes normal complete blood count and comprehensive metabolic panel. Serum calcium, parathyroid hormone level and TSH were within normal limits. Her 24-h urine catecholamines were elevated: metanephrines 683 mg/24 h (normal range: 30 - 180 mg/24 h), normetanephrines 6,391 mg/24 h (normal range: 148 - 560 mg/24 h), total metanephrines 7,074 mg/24 h (normal range: 180 - 646 mg/24 h), epinephrine 11 mg/24 h (normal range: < 21 mg/24 h), norepinephrine 151 mg/24 h (normal range: 15 - 80 mg/24 h) and dopamine 605 mg/24 h (normal range: 65 - 400 mg/24 h). Plasma normetanephrines (free) was 18 nmol/L (normal range: < 0.09 nmol/L) and chromogranin A, S was 527 ng/mL (normal range: < 93 ng/mL). Magnetic resonance imaging (MRI) of the abdomen and pelvis revealed metastatic disease in bilateral proximal femurs, vertebrae and iliac bones (Fig. 2, 3). Patient was also tested for RET mutation, and was found to be negative for the same.

**Figure 4:**
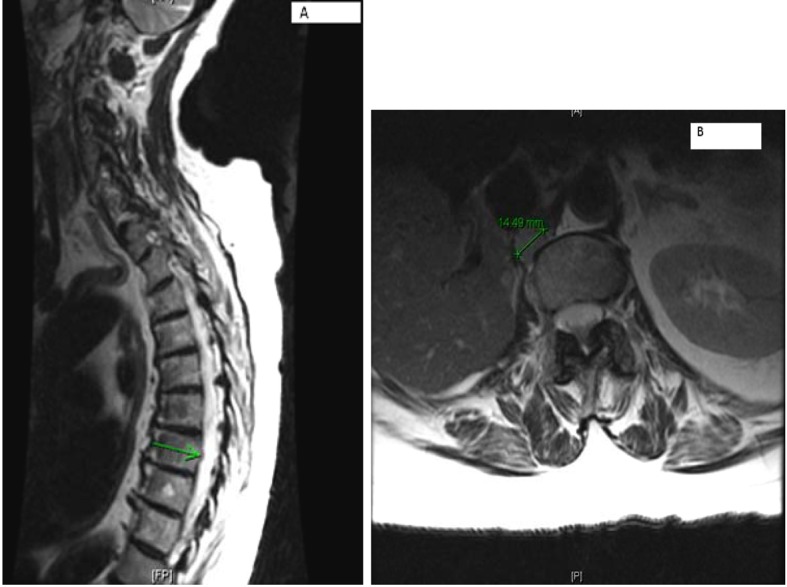
(A) Multilevel-multifocal osseous metastases particularly within thoracolumbar spine with slight epidural extension at T-7 and mild epidural disease at T-10 without cord compression. (B) Right retrocrural/paraspinal metastasis is seen at T-12 and L-1 level measuring about 1.5 cm.

MRI of the spine confirmed bilateral iliac bone metastasis, T-10 diffuse infiltration of metastasis, retroperitoneal lymph nodes and several nodules along the crus of the right diaphragm (Fig. 4). Patient was started on denosumab and zoledronic acid, and she received 10 fractions of radiotherapy to her right hip.

**Figure 5:**
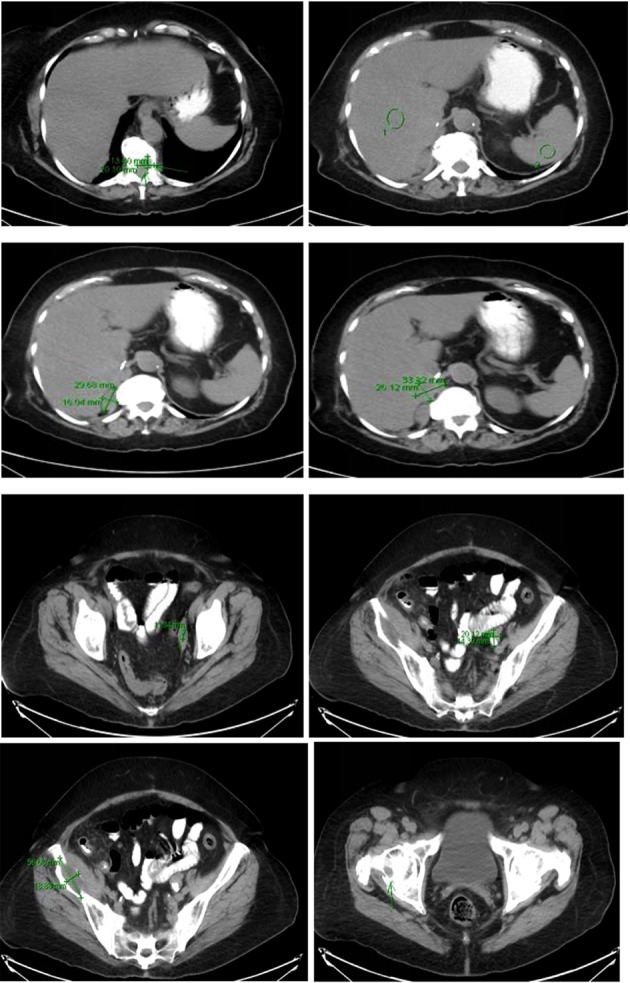
Multiple predominantly osseous metastasis with metastasis in the right iliac bone, increased conspicuity of several soft tissue nodules in the right adrenalectomy bed consistent with recurrent neoplasm, a superior structure measuring 3.4 × 2.6 cm not well defined from adjacent unopacified liver is seen, the right diaphragmatic crus and the unopacified IVC and an adjacent posterior nodule measuring 3 × 1.6 cm is noted.

Computer tomography of the abdomen also showed increased soft tissue nodules in the right adrenal bed with multiple lytic osseous lesions, and distal ileal wall thickening secondary to post radiotherapy changes (Fig. 5).

In view of her extensive metastatic disease, patient was offered a metaiodobenzylguanidine scan (MIBG), and positive subsequent treatment with 131- radioactive iodine was discussed with her. However, patient denied any further intervention and was made hospice.

## Discussion

Paragangliomas are rare neuroendocrine tumors arising in both sympathetic and parasympathetic paraganglia. Eighty percent of these tumors arise from the adrenal medulla and are referred to as PCCs. Approximately 20% of these tumors are located in extra-adrenal sites and are referred to as secreting paragangliomas. Tumors arising from the parasympathetic tissue in the head and neck region are included in the second category. Usually the PCCs and the paragangliomas arising in the abdomen produce catecholamines [1].

Hypertension is the most common feature of PCCs and abdominal paragangliomas [1]. However, head and neck paragangliomas are usually silent, but can cause mass effect and local infiltration of the adjacent structures. Dysphagia, tinnitus and cranial nerve palsies have been reported with head and neck paragangliomas [2].

Approximately 10% of PCCs are malignant [3]. Malignant PCCs and paragangliomas are extremely rare tumors. In 2002, in the United States, the estimated incidence of malignant paragangliomas was 93 cases per 400 million persons [4]. Metastatic spread can occur at presentation or years after primary diagnosis. Malignant PCCs and paragangliomas secrete predominately noradrenaline but can also exclusively secrete dopamine. A very high plasma chromogranin A level is usually seen in patients with malignant PCCs [5]. However, falsely elevated levels of chromogranin A have been seen in patients with liver and kidney disease, and with the use of proton pump inhibitors [6]. Malignant PCCs are also associated with high plasma levels of neuron specific enolase [7].

MIBG has same chemical properties as norepinephrine, and is concentrated in chromaffin tissue via the human norepinephrine transporter. Therefore, a whole body MIBG scan using I^123^ or I^131^ can be used to evaluate the extent of extra-adrenal disease. The sensitivity and specificity of MIBG scan are 83-100% and 95-100%, respectively [8]. However, patients with mutations in genes responsible for succinate dehydrogenase subunits and those with exclusive dopamine secreting paragangliomas are usually not good candidates for an MIBG scan [9]. In these patients, other tracers can be used. Indium-11-DTPA-octreotide is the most common tracer used in patients with a negative MIBG scan. Somatostatin analogues labeled with gallium-68 have been used in PET imaging. Radiolabeled dopamine or dihydroxyphenylalanines have also been used as tracers in PET imaging.

In general, paragangliomas larger than 5 cm, extra-adrenal location and the presence of necrosis are associated with a higher risk of malignancy. Thompson proposed a “PCC of the adrenal gland scales score” (PASS), which can be used to evaluate the malignant potential of a PCC. A PASS score of < 4 is considered to be benign, where as a score of > 6 is usually suggestive of a malignant paraganglioma (Table 1) [10].

**Table 1:** Pheochromocytoma of the Adrenal Gland Scoring Scale (PASS)

Nuclear hyperchromasia	1
Profound nuclear pleomorphism	1
Capsule invasion	1
Vascular invasion	1
Extension into adipose tissue	2
Atypical mitotic figures	2
Greater than 3 of 10 mitotic figures high-power field	2
Tumor cell spindling	2
Cellular monotony	2
High cellularity	2
Central or confluent tumor necrosis	2
Large nests or diffuse growth (> 10% of tumor volume)	2
Total	20

Recently, various molecular markers have been associated with PCCs and malignant paragangliomas. These include secretogranin II-derived peptide, N-cadherin, vascular endothelial growth factor (VEGF) and endothelian receptors. Overexpression of the telomerase catalytic subunit and the heat shock protein 90 (HSP90) have also been associated with malignant paragangliomas [11]. In a recent study by Meyer-Rochow et al, 29 adrenal medulla samples were studied. Overexpression of mi-483-5p and underexpression of miR15a and miR-16 (involved in apoptosis) were noted in malignant PCCs [12]. A Ki-67 index of > 3% can also be used for predicting the malignant potential of a paraganglioma [13].

PCCs and other paragangliomas can either be sporadic or hereditary in origin. Hereditary syndromes associated with paragangliomas include von Hippel Lindau disease (VHL), multiple endocrine neoplasia type 2 and neurofibromatosis type 1. Recently mutations in genes responsible for four subunits of succinate dehydrogenase (A-D) have been identified. These have been associated with syndromes named paragangliomas 1-5 [14]. Mutations in genes responsible for succinate dehydrogenase subunit B are generally associated with a poor prognosis [15]. Various genes have been associated with paragangliomas. These genes are either associated with an abnormal activation of the tyrosine kinase signaling pathway or activation of the mammalian target of rapamycin (m-TOR) signaling pathway.

Surgical resection is the mainstay of treatment for PCCs and other malignant paragangliomas. The overall 5-year survival rate for malignant paragangliomas ranges from 30 to 60% [16]. Patients with unresectable metastatic disease are considered candidates for radiometabolic treatment. I^123^ MIBG, I^131^ MIBG and radiolabeled somatostatin analogues have been used successfully for the ablation of metastatic disease [17, 18]. Side effects include thrombocytopenia and rarely bone marrow toxicity. External radiotherapy is mainly of palliative use, to relieve pain secondary to bony metastasis. Chemotherapy is reserved for patients with unresectable disease and for lesions resistant to radioablation [19]. Although a combination of cyclophosphamide, vincristine and dacarbazine (CVD) has been used for treating neuroblatomas, their role in treating malignant PCCs still needs to be validated. In a trial recently updated by National Institutes of Health, 14 case of malignant PCCs treated with CVD were followed up over a period of 22 years. Symptom relief and tumor regression were seen in 50% of the patients. However, no significant change in overall survival was noted [20].

With better understanding of the molecular pathways involved in malignant PCCs and other paragangliomas, various new therapies have been introduced in recent years. In general, both benign and malignant PCCs are associated with gene mutations that have been broadly classified into two categories. The first category includes mutations in VHL and succinate dehydrogenase B and D genes. These are associated with abnormal VEGF signaling and overexpression of angiogenic factors. The second category includes mutations in RET, NF1, TMEM127 and MAX genes. These are associated with abnormal signaling of the tyrosine kinase and the m-TOR1 pathway. Therefore, use of drugs targeting these abnormal pathways seems to be a promising option for treating these rare tumors in future. Sunitinib, a tyrosine kinase inhibitor, has been successfully used for treating malignant PCCs [21, 22]. Park et al reported sunitinib as an effective option for treating malignant paragangliomas in patients resistant to anthracyclines and platinum-based chemotherapy [23].

Thalidomide, by targeting the VEGF signaling pathway and by virtue of its antiangiogenic properties, has been used successfully for treating various malignancies. A combination of thalidomide and temozolomide has been used for treating malignant PCCs and other neuroendocrine tumors [24]. Lastly, overexpression of HSP90 (chaperon involved in folding and stabilizing various oncoproteins) has been associated with malignant PCCs and other paragangliomas. Therefore, targeting this abnormality seems to be a promising treatment option for malignant PCCs and other paragangliomas in future [11, 25-26].

### Conclusion

Malignant PCCs are extremely rare tumors with a very poor prognosis. Abnormal signaling involving the tyrosine kinase, m-TOR and VEGF pathways has been associated with a higher risk of malignancy. Molecular targeted therapy against these abnormal pathways seems to be a promising option for treating these aggressive tumors in future. However, extensive research is required, before these treatment options are widely acceptable.
